# Factors affecting quantity of pollen dispersal of spray cut chrysanthemum (*Chrysanthemum morifolium*)

**DOI:** 10.1186/1471-2229-14-5

**Published:** 2014-01-06

**Authors:** Xiao-Guang Wang, Hai-Bin Wang, Fa-Di Chen, Jia-Fu Jiang, Wei-Min Fang, Yuan Liao, Nian-Jun Teng

**Affiliations:** 1 College of Horticulture, Nanjing Agricultural University, Nanjing 210095, China; 2 Jiangsu Province Engineering Lab for Modern Facility Agriculture Technology & Equipment, Nanjing 210095, China

## Abstract

**Background:**

Spray cut chrysanthemum is a vital flower with high ornamental value and popularity in the world. However, the excessive quantity of pollen dispersal of most spray cut chrysanthemum is an adverse factor during its flowering stage, and can significantly reduce its ornamental value and quickly shorten its vase life. More seriously, excessive pollen grains in the air are usually harmful to people, especially for those with pollen allergies. Therefore, in order to obtain some valuable information for developing spray cut chrysanthemum with less-dispersed or non-dispersed pollen in the future breeding programs, we here investigated the factors affecting quantity of pollen dispersal of spray cut chrysanthemum with four cultivars, i.e. ‘Qx-097’, ‘Noa’, ‘Qx-115’, and ‘Kingfisher’, that have different quantity of pollen dispersal.

**Results:**

‘Qx-097’ with high quantity of pollen dispersal has 819 pollen grains per anther, 196.4 disk florets per inflorescence and over 800,000 pollen grains per inflorescence. The corresponding data for ‘Noa’ with low quantity of pollen dispersal are 406, 175.4 and over 350,000, respectively; and 219, 144.2 and nearly 160,000 for ‘Qx-115’ without pollen dispersal, respectively. ‘Kingfisher’ without pollen dispersal has 202.8 disk florets per inflorescence, but its anther has no pollen grains. In addition, ‘Qx-097’ has a very high degree of anther cracking that nearly causes a complete dispersal of pollen grains from its anthers. ‘Noa’ has a moderate degree of anther cracking, and pollen grains in its anthers are not completely dispersed. However, the anthers of ‘Qx-115’ and ‘Kingfisher’ do not crack at all. Furthermore, microsporogenesis and pollen development are normal in ‘Qx-097’, whereas many microspores or pollen degenerate in ‘Noa’, most of them abort in ‘Qx-115’, and all of them degrade in ‘Kingfisher’.

**Conclusions:**

These results suggest that quantity of pollen dispersal in spray cut chrysanthemum are mainly determined by pollen quantity per anther, and capacity of pollen dispersal. Abnormality during microsporogenesis and pollen development significantly affects pollen quantity per anther. Capacity of pollen dispersal is closely related to the degree of anther dehiscence. The entire degeneration of microspore or pollen, or the complete failure of anther dehiscence can cause the complete failure of pollen dispersal.

## Background

Chrysanthemum (*Chrysanthemum morifolium* (Ramat.) Kitamura) is among the ten most popular traditional flowers in China and one of the four most popular cut flowers in the world. Thus, this species occupies a vital position in production of flowers due to its high ornamental value [1,2]. Spray cut chrysanthemum is a type of chrysanthemum important in chrysanthemum production. It has become the most important cut flower in China, and the second largest type of cut flowers in the United States and Europe, due to richness in floral colors and shapes, uniform flowering, and plentiful spray flowers as well [3,4]. However, the ornamental value and vase life of spray cut chrysanthemum usually drop with the increase in quantity of pollen dispersal of middle tubular bisexual flowers at the flowering stage. More seriously, plenty of pollen grains produced by spray cut chrysanthemum at the flowering stage will float in the air and may cause severe allergic reactions that could be harmful to people, in particular those with pollen allergies [5-7]. Therefore, it is very necessary and urgent to solve the problem of pollen contamination during flowering of spray cut chrysanthemum.

**Figure 1:**
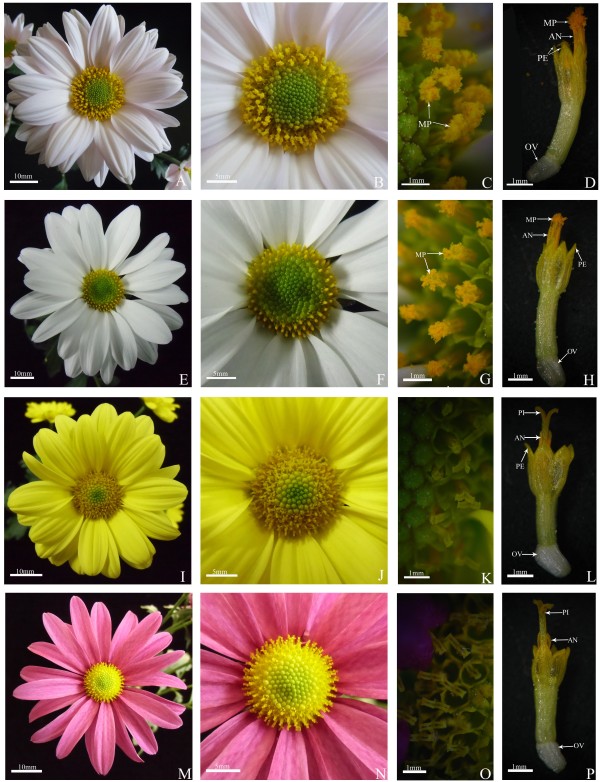
**Flower morphology of four spray cut chrysanthemum cultivars.** ‘Qx-097’ **(A-D)**, ‘Noa’ **(E-H)**, ‘Qx-115’ **(I-L)**, and ‘Kingfisher’ **(M-P). (A, B, E, F, I, J, M, N)** Inflorescence morphology. **(C, D, G, H, K, L, O, P)** Disk floret. AN: Anther; MP: Mass of Pollen Grains; OV: Ovule; PE: Petal; PI: Pistil.

Similar problem also exists in production of cut lily flowers. In order to reduce adverse effects of pollen contamination, and improve ornamental value and vase life of lily, producers usually remove anthers from cut lily flowers artificially just after their harvest when the anthers have not started to disperse pollen [8-10]. Although this is time-consuming, laborious and increases product cost a little, the advantages of this measure outweighs its disadvantages as a whole. This measure is feasible in lily and even some other cut flowers with large anthers, but not feasible in spray cut chrysanthemum. The main reason is that inflorescence structure of spray cut chrysanthemum is not suitable for artificial removal of anther or emasculation, each chrysanthemum inflorescence consists of 20-30 peripheral ray florets with only pistil and 100-200 small central disk florets with both pistil and stamen (Figure 1). Thus it is nearly impossible to artificially remove the anthers. Even producers can emasculate the small central disk florets, but such emasculation will make the spray cut chrysanthemum lose the ornamental value immediately [1]. Therefore, the best way to solve the problems of pollen dispersal during flowering of spray cut chrysanthemum is to develop new cultivars with less-dispersed or non-dispersed pollen through breeding programs.

It will be very useful to have some important information on factors influencing quantity of pollen dispersal of spray cut chrysanthemum before starting to develop new cultivars that disperse less pollen or do not disperse pollen at all through breeding methods. However, such information is not available by now. Therefore, we are the first time to carry out a systematic investigation on factors controlling quantity of pollen dispersal of spray cut chrysanthemum in this study using four cultivars with different quantity of pollen dispersal, i.e. ‘Qx-097’ with high quantity of pollen dispersal, ‘Noa’ with low quantity of pollen dispersal, ‘Qx-115’ and ‘Kingfisher’ without pollen dispersal. Our overall aim was to reveal the main causes influencing quantity of pollen dispersal of spray cut chrysanthemum, and the expected outputs will provide valuable information for effectively developing new cultivars with less-dispersed or non-dispersed pollen in the near future.

## Methods

### Experimental materials

According to our previous observations on morphological characteristics of pollen dispersal, four spray cut chrysanthemum cultivars with different quantity of pollen dispersal were screened and grown in the Chrysanthemum Germplasm Resource Preserving Center, Nanjing Agricultural University, China (32°05’ N, 118°90’ E). They were ‘Qx-097’ with high quantity of pollen dispersal, ‘Noa’ with low quantity of pollen dispersal, ‘Qx-115’ and ‘Kingfisher’ without pollen dispersal, respectively (Figure 1).

### Determination on pollen quantity per inflorescence

Because each chrysanthemum inflorescence usually consists of 20-30 peripheral ray florets which contain only a pistil and 100-200 small central disk florets with both pistil and stamen, thus only central disk florets can produce pollen and disperse pollen [1]. Therefore, ten inflorescences at the full flowering stage of each cultivar were randomly sampled for determining the number of central disk florets per inflorescence. In addition, ten central disk florets were randomly selected for determining the number of anther per disk floret. Furthermore, 60 anthers just before dehiscence were randomly sampled from each cultivar for estimating the number of pollen per anther. The anthers were put in a 10 ml centrifuge tube, and then stored at 50°C for around 24 hours. Afterwards, 6 ml of 20% (NaPO_3_)_6_ solution (w/v) was added into the centrifuge tube, and then the tube was shaken and inverted completely to produce pollen suspension. 2 μl of pollen suspension was added on a hemocytometer and pollen amount was counted under an Olympus BX41 microscope. Pollen quantity per anther was calculated with the formula [pollen quantity per anther (pollen grains/anther) = the number of pollen grains in 2 μl suspension × 3000/60]. Each experiment was repeated six times. Pollen quantity per inflorescence was calculated according to pollen quantity per anther, number of anthers per disk floret, and number of disk florets per inflorescence, i.e. pollen quantity per inflorescence = pollen quantity per anther × number of anthers per disk floret × number of disk florets per inflorescence.

### Determination on pollen dispersal

At the full flowering stage of each cultivar, the images of pollen on stigmas were captured for the purpose of assessing morphological characteristics of pollen dispersal. In addition, the anthers after their pollen dispersal were collected for determining the level of anther cracking and the amount of pollen left per anther by the above-mentioned method. Furthermore, some of the anthers after their pollen dispersal were subject to the following microscopy technology.

### Microsporogenesis and pollen development

Microsporogenesis and pollen development of the four cultivars were examined according to the paraffin section method of [11,12] with some modifications. Flower buds and disk florets of each cultivar at different development stages were collected, and immediately immersed in FAA solution (formalin 5 ml, acetic acid 5 ml, alcohol 70% 90 ml) until use. The samples were dehydrated through a graded series of ethanol solutions, and then embedded in paraffin wax. Sections were cut to a thickness of 8 μm and stained with Heidenhain’s hematoxylin. Then the sections were observed and imaged under an Olympus BX41 microscope. Digital images were captured using an Axiocam MRC camera.

In addition, the anthers of ‘Qx-097’ and ‘Noa’ at different development stages were also subject to transmission electron microscopy (TEM) by [13,14] with some modifications. Fresh anthers were stripped from central disk florets and immediately immersed in 2.5% (v/v) glutaraldehyde (in 0.1 mol/L phosphate buffer, pH 7.2), gently expelled using a syringe, and then stored at 4°C until use. Then the anthers were washed five times with the same phosphate buffer and post-fixed in 1.5% osmium tetroxide for 5 h. Afterwards, they were treated through a graded series of PHEM buffer (60 mmol/L pipes; 25 mmol/L Hepes; 10 mmol/L EGTA; 2 mmol/L MgCl_2_; pH 7. 0) and ethanol solutions, and then embedded in Epon 812. Sections were cut to a thickness of 80 nm using an LKB-V ultra-microtome (Bromma, Sweden) and stained with uranyl acetate and lead citrate. The sections then were observed and imaged under a transmission electron microscope (Hitachi H-7650) at 80 kV.

### Statistical analysis

The data were subjected to a one-way analysis of variance using the SPSS software 16.0 (SPSS Inc, Chicago, IL, USA), and the means were compared using the Bonferroni t-test with alpha = 0.05.

## Results

### Pollen quantity per inflorescence

The number of disk florets per inflorescence of ‘Qx-097’ , ‘Noa’ , ‘Qx-115’ and ‘Kingfisher’ are 196.4, 175.4, 144.2, and 202.8, respectively (Table 1). All the four cultivars have the same number of anthers per floret. However, significant differences are observed on pollen quantity per anther and inflorescence among the four cultivars (Table 1; Figure 1). For instance, ‘Qx-097’ has the most pollen grains per anther and inflorescence that are 819 and over 800,000, respectively, which is further supported by the amount of pollen grains on stigmas after anther dehiscence (Figure 1). However, ‘Kingfisher’s inflorescence does not contain any pollen grains, for its anthers fail to produce pollen grains. These results indicate that pollen quantity per anther is a main factor affecting pollen quantity per inflorescence of spray cut chrysanthemum, while the number of disk florets per inflorescence has little effect on pollen quantity per inflorescence.

**Table 1:** Inflorescence traits of four spray cut chrysanthemum cultivars

**Cultivar**	**Disk florets per inflorescence**	**Anthers per floret**	**Pollen quantity per anther**	**Pollen quantity per inflorescence**	**Apparent pollen quantity**	**Anther dehiscence degree**	**Percentage of pollen dispersal (%)**
Qx-097	196.4 ± 9.0ab	5	819 ± 30a	804258a	Much	High	Nearly 100
Noa	175.4 ± 2.9b	5	406 ± 31b	356062b	Less	Medium	Over 50
Qx-115	144.2 ± 7.0c	5	219 ± 19c	157899c	None	Non-cracking	0
Kingfisher	202.8 ± 9.3a	5	0d	0d	None	No pollen in anther	0

Values (mean ± standard error) with the same letter are not significantly different at a = 0.05 by the Bonferroni t-test using SPSS16.0.

### Pollen dispersal per inflorescence

The apparent pollen quantity is a morphological parameter that grossly indicates the amount of pollen grains gathering on stigmas during anther dehiscence of spray cut chrysanthemum. In other words, this parameter is an intuitive reflection of quantity of pollen dispersal, or can be regarded as a criterion assessing the capacity of chrysanthemum pollen dispersal. For example, mass of pollen grains on stigmas of ‘Qx-097’ during anther dehiscence were clearly visible and large in volume (Figure 1A-D), so ‘Qx-097’ is a cultivar with high quantity of pollen dispersal. There are also mass of pollen grains on stigmas of ‘Noa’ during anther dehiscence, but pollen mass is much smaller in volume compared with that of ‘Qx-097’ (Figure 1E-H). Thus, ‘Noa’ is considered as a chrysanthemum cultivar with low quantity of pollen dispersal. ‘Qx-115’ does not disperse pollen during anther dehiscence and no any pollen grains can be observed on its stigmas (Figure 1I-L). Therefore, ‘Qx-115’ is regarded as a cultivar without pollen dispersal, although it has 219 pollen per anther and nearly 160,000 per inflorescence (Table 1). ‘Kingfisher’ does not dispersal pollen at all during anther dehiscence (Figure 1M-P), as its anther or inflorescence does not contain any pollen (Table 1), thus it is a cultivar with non-dispersed pollen.

### Microsporogenesis and pollen development of ‘Qx-097’

Because ‘Qx-097’ is a cultivar with high quantity of pollen dispersal, thus the detailed reproductive processes of microsporogenesis and pollen development were presented here, which will provide some useful information for revealing the reason of its high quantity of pollen dispersal from reproductive aspect.

**Figure 2:**
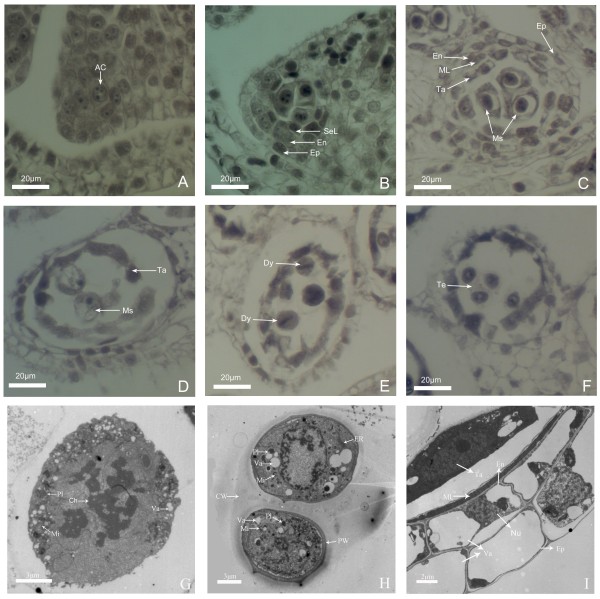
**Microsporogensis of ‘Qx-097’ by TEM and paraffin section method. (A)** Differentiation of archesporial cells. **(B)** Microsporocyte formation stage, showing differentiation of microspore mother cell and 3 anther walls (secondary parietal cell layer, endothecium and epidermis from the inner to the outer layer). **(C)** Microsporocyte formation stage, showing microspore mother cell tightly abutted and four complete anther walls. **(D)** Microspore mother cell at prophase I of meiosis, showing microspore mother cell in oval shape, discohesive cells and prominent nucleoli. **(E)** Microspores at first meiotic metaphase, chromosomes aligned on the metaphase plate. **(F)** Tetrad and condensed cytoplasm of tapetal layer. **(G)** Microspore mother cell in pachytene, showing mitochondria, plastids and numerous vacuoles. **(H)** The stage of tetrad, showing mitochondria, plastids, endoplasmic reticulum, enlargement of vacuoles, callose wall and cell wall which begin to develop. **(I)** Anther wall at microsporocyte meiosis stage, cytoplasm of endothecium layer, epidermal layer and tapetal layer cells become vacuolation, cytoplasm of tapetal layer cells which has large nuclei and obvious nucleoli condensed, middle layer degrades. AC: Archesporial cells; Ch: chromatin; CW: Callose wall; Dy: Dyad; En: Endothecium; Ep: Epidermis; ER: Endoplasmic reticulum; Mi: Mitochondrion; ML: Middle layer; Ms: Microspore mother cell; Nu: Nucleus; Pl: Plastid; PW: Pollen wall; SeL: Secondary parietal cell layer; Ta: Tapetum; Te: Tetrad; Va: Vacuole.

Microsporogenesis includes the periods from archesporial cell differentiation to tetrad formation (Figure 2). At the beginning of development, transverse section of anther was trapezoid in shape, and archesporial cells were differentiated at the four corners of the anther (Figure 2A). As development proceeded, the archesporial cells first developed into primary sporogenous cell and primary parietal cells layer through periclinal division, and the primary parietal cells continued to divide into secondary parietal cells layer and endothecium (Figure 2B). After that, secondary parietal cells divided into tapetum and middle layer. Meanwhile primary sporogenous cells were also in a series of differentiation and finally microspore mother cells and the entire anther walls were formed (Figure 2C). The microspore mother cells were interconnected and characterized by a large, centrally located nucleus. The anther walls consisted of four layers from the inner to the outer layers: tapetum, middle layer, endothecium, and epidermis. With the further development of anther, the microspore mother cells underwent meiosis and reached to the prophase I of meiosis. At this time, the nuclei of microspore mother cells showed chromonema and prominent nucleoli (Figure 2D). In addition, the microspore mother cells contained plenty of electron-dense cytoplasm, lots of mitochondria and plastids, and many vacuoles near the plasma membrane (Figure 2G). The microspore mother cells gave rise to tetrahedral tetrads with four haploid microspores (Figure 2F) after undergoing metaphase I and dyad stage (Figure 2E). The microspores cells contained abundant ribosome and endoplasmic reticulum, mitochondria, plastids as well as vacuoles, and microspore wall or pollen wall can be observed at late tetrad stage (Figure 2H). Meanwhile, middle layer cells have begun to degrade, and the cytoplasm in endothecium and epidermal cells nearly disappeared, but the cytoplasm in tapetal cells are condensed and stained very dark (Figure 2I).

**Figure 3:**
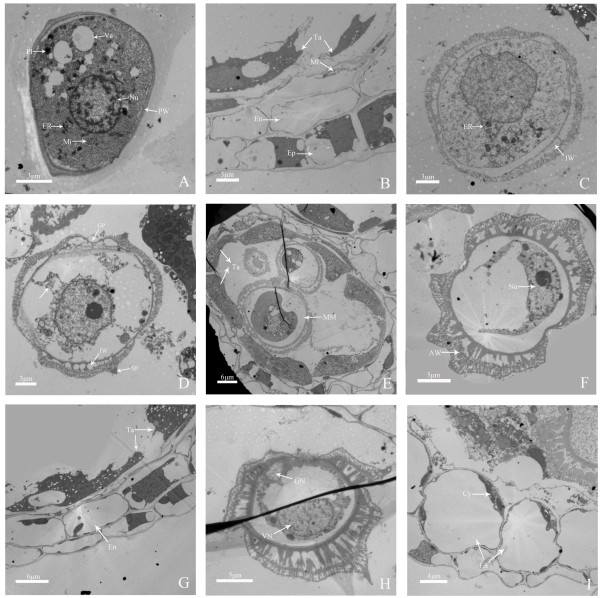
**Pollen development of ‘Qx-097’ by TEM. (A)** Early microspore, microspore cells have uniform and dense cytoplasm, abundant endoplasmic reticulum, mitochondria, plastids, some growing vacuoles and a nucleus which is in center of cells. **(B)** Anther wall at the early microspore stage, vacuolation of cytoplasm of tapetal layer with discohesive cells increase, middle layer is in further degradation. **(C-D)** Middle microspore, showing inner wall which begin to develop, spiked protuberances appear on the outside of the walls, recognizably germ pores, enlargement of vacuoles, off-centre nuclei and degraded cytoplasm and the number of density of ribosome, mitochondria and plastids decrease. **(E)** At middle microspore stage, tapetal cells contained abundant cell organelles and dense cytoplasm. **(F)** Late microspore stage, microspore cell wall, especially inner wall thickens, large central vacuole forms and squeeze the cytoplasm and nuclei to against the wall. **(G)** Late microspore stage, tapetal layer cells are hill-shaped and inner tangential walls are almost degraded completely, middle layer almost disappear and only has a few residue. **(H)** Early bicellular pollen stage, there is develop inner wall, exine formation by deposition of primexine and then sporopollenine, a large central vegetative cell and a small generative cell which forms by excentrically division of microspores nuclei. **(I)** Early bicellular pollen stage, tapetal layer almost disappear, endothecium layer cells enlarge remarkably in volume. Cy: Cytoplasm; En: Endothecium; Ep: Epidermis; ER: Endoplasmic reticulum; GN: Generative nucleus; GP: Germ pore; IW: Inner wall of cells; Mi: Mitochondrion; Ml: Middle layer; MM: Middle microspore; Nu: Nucleus; Pl: Plastid; PW: Pollen wall; SP: Spiked protuberance; Ta: Tapetum; Va: Vacuole; VN: Vegetative nucleus.

**Figure 4:**
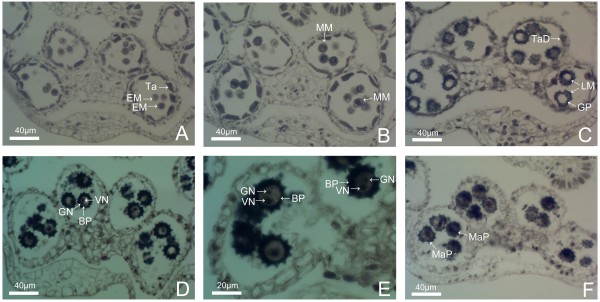
**Pollen development of ‘Qx-097’ by paraffin section method. (A)** Early microspores, tapetal layer cells condensed. **(B)** Middle microspores stage, showing germ pores, thickening cell walls and radial thinning of tapetal layer cells. **(C)** Late microspores stage, showing mononuclear microspores against the anther wall and degradation of tapetal layer. **(D)** Early bicellular pollen stage, showing nuclei divide excentrically to form a large central vegetative nucleus and a small generative nucleus, the tapetal cells only have residue and endothecium layer cells enlarge in volume. **(E)** Late bicellular pollen stage, contents such as starch began to accumulate in cytoplasm, but the two nuclei can be observed still and vacuoles gradually disappeared. **(F)** Mature pollen stage, contents such as starch fills the whole cell and the nuclei are hard to be observed. BP: Bicellular pollen; EM: Early microspore; GN: Generative nucleus; GP: Germ pore; LM: Late microspore; MaP: Mature pollen; MM: Middle microspore; Ta: Tapetum; TaD: Tapetum debris; VN: Vegetative nucleus.

After the release of haploid microspores from the tetrads, microspores started to undergo mitosis and finally form bicellular pollens (Figures 3 and 4). At early microspore stage, the young microspore has one conspicuous, centrally located nucleus, dense cytoplasm, a thin cell wall, lots of ribosome endoplasmic reticulum, mitochondria, plastids, and some growing vacuoles (Figures 3A and 4A). In addition, middle layer cells had almost degenerated, endothecium cells became completely empty (Figure 3B). At middle microspore stage, cell walls and germ pores can be observed, spiked protuberances appeared on the outer surface of cell walls, and the number of ribosome, mitochondria and plastids decreased, whereas several vacuoles were formed and occupied much space (Figures 3C, D and 4B). Tapetal cells were thin, but contained abundant cell organelles such as endoplasmic reticulum, mitochondria, and plastids (Figure 3E). At late microspore stage, nuclei and cytoplasm were squeezed by one large central vacuole and migrated from the center toward the cell periphery, and cell walls increased in thickness (Figures 3F and 4C). Tapetal cells were hill-shaped and their inner tangential walls were almost degraded, and middle layer cells nearly disappeared (Figure 3G). At the early bicellular pollen stage, microspore nucleus divided to form a large vegetative cell and a small generative cell against the outer wall, the tapetal cells have nearly disappeared, and the endothecium cells enlarged in volume (Figures 3H, I and 4D). Afterwards, the generative cell moved away from the wall and began to move near the vegetative cell; contents such as starch began to accumulate in cytoplasm, but the two nuclei can be observed still and vacuoles gradually disappeared (Figure 4E). When pollen grain became mature, the contents such as starch nearly filled the whole cytoplasm in pollen grain, and it is hard to observe vacuoles in cytoplasm (Figure 4F).

### Microsporogenesis and pollen development of ‘Noa’, ‘Qx-115’ and ‘Kingfisher’

**Figure 5:**
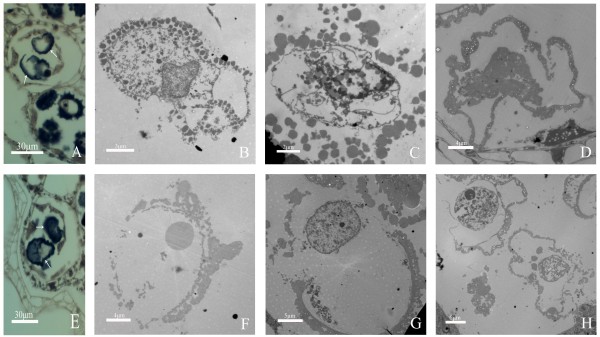
**Pollen abortion during pollen development of ‘Noa’. (A)** Late microspore stage, showing rupture of cell wall and disintegration of cytoplasm (arrows). **(B)** Early microspore stage, showing rupture of the primary cell wall, shrinkage of cytoplasm and the nucleus which was in irregularly shaped. **(C)** Microspore in early stage of development, cell wall and cytoplasm were in degradation. **(D)** Distortion of cell wall of microspore and shrinkage of cytoplasm. **(E-H)** Pollen abortion in late pollen development stage (arrows).

**Figure 6:**
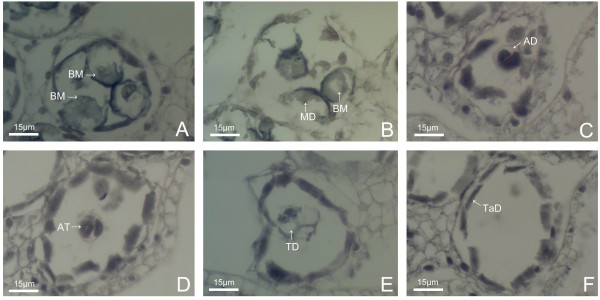
**Pollen abortion during pollen development of ‘Qx-115’ and ‘Kingfisher’.** ‘Qx-115’ **(A-B)** and ‘Kingfisher’ **(C-F). (A)** Microspores at middle microspores stage of ‘Qx-115’, showing many broken, incomplete and abortive microspores. **(B)** Late microspores stage in ‘Qx-115’, it has few complete pollen grains in the anther. **(C)** Asymmetrical and abnormal dyad. **(D)** Asymmetrical and abnormal tetrad. **(E)** Degradation of tetrads, microspores can’t move apart and cytoplasm was degraded and in vacuolization. **(F)** Early microspores stage, showing pollen sac with no pollen grains of ‘Kingfisher’. AD: Abnormal dyad; AT: Abnormal tetrad; BM: Broken microspore; MD: Microspore in degradation; TaD: Tapetum debris.

Microsporogenesis and pollen development of ‘Noa’ , ‘Qx-115’ and ‘Kingfisher’ are similar to that of ‘Qx-097’. Abnormalities were seldom observed during microsporogenesis and pollen development of ‘Qx-097’ , but ‘Noa’ , ‘Qx-115’ and ‘Kingfisher’ had a high percentage of abnormalities (Figures 5 and 6; Table 2). For ‘Noa’ , many pollen grains were abortive after the release of microspores from tetrads (Figure 5A-H). Similarly, there were many broken, incomplete pollen grains in anthers at the middle microspore stage of ‘Qx-115’ (Figure 6A), and few complete pollen grains in the late microspore stage (Figure 6B). Therefore, degeneration of a high proportion of pollen grains may be the main reason that pollen quantity per anther of ‘Noa’ and ‘Qx-115’ was lower than that of ‘Qx-097’ (Table 1). For ‘Kingfisher’, a very high percentage of abnormalities occurred at the microsporocyte meiosis stage, which is the main reason responsible for no pollen grains in anthers of this cultivar. For example, at the dyad or tetrad stage, only a few dyads or tetrads were observed in microsporangia of anther, and most of them were in degeneration (Figure 6C-E). Afterwards, no pollen grains were observed in microsporangia of anther (Figure 6F). The results presented here indicate that pollen quantity of spray cut chrysanthemum cultivars is closely related to the degrees of abnormalities during microsporogenesis and pollen development.

**Table 2:** Comparisons of microsporogenesis, pollen development and pollen dispersal among four spray cut chrysanthemum cultivars

**Stage**	**Major events in pollen development and pollen dispersal of ‘Qx-097’**	**The differences of ‘Noa’, ‘Qx-115’ and ‘Kingfisher’ compared with ‘Qx-097’**
Microsporocyte formation stage	Tissue differentiation, microspore mother cells in irregular shape, tightly abutting, relatively large nuclei, formation of 4 complete anther walls.	None.
Microsporocyte meiosis stage	Oval-shaped microspore mother cells, meiosis, microspores in tetrahedral tetrads, microspore mother cells and tetrads surrounded by callose and abundant cytoplasm contains mitochondria, plastids, endoplasmic reticulum, small vacuoles, vacuolation of cytoplasm of endothecium layer and epidermal layer cells, condensed cytoplasm of tapetal layer cells.	‘Kingfisher: asymmetrical dyad and tetrad formed by inequality meiosis, degradation and vacuolization of microspore cytoplasm in tetrad, abnormal degradation and microspores not released from tetrads.
Early microspore stage	Free microspores released from tetrads, no vacuoles, no germ pores, thin cell wall, uniform and dense cytoplasm, nuclei in center of cells. vacuolation of cytoplasm of tapetal layer with discohesive cells increase, middle layer in further degradation;	‘Kingfisher’: nothing in pollen sacs, pollen abortion completely;
		‘Noa’: individual microspore abortion.
Middle microspore stage	Microspore enlargement, germ pores formation, cells wall thickenings formed, spiked protuberances formed and inner wall formed, vacuoles increase and enlargement, degradation of microspore cytoplasm, off-centre of nuclei, tapetal layer cells radial thinning.	‘Qx-115’: large number of microspore abortion, cells broken, degradation of cytoplasm;
		‘Noa’: individual microspore abortion;
		‘Kingfisher’: nothing in pollen sacs.
Late microspore stage	Cell walls thickened and cells enlargement continues, large central vacuole formation, nuclei and cytoplasm at opposite side of germ pores against outer walls, tapetal layer cells in hill-shaped and further degradation, middle layer almost disappeared.	‘Qx-115’: microspore abortion continues, few complete pollen grains in the late microspore stage;
		‘Noa’: individual microspore abortion;
		‘Kingfisher’: nothing in pollen sacs.
Early bicellular pollen stage	Inequality mitosis of microspore, vegetative cell and generative cell formation, remains of tapetal layer, endothecium layer cells enlargement.	Anthers start shriveling of ‘Qx-115’ and ‘Kingfisher’.
Late bicellular pollen stage	Bicellular pollen stage enlargement, move of generative cell to vegetative cell, starch accumulation, ‘U’ shaped thickened of endothecium layer cell walls.	Anthers shriveled of ‘Qx-115’ and ‘Kingfisher’.
Mature pollen stage	The contents like starch fill the whole cytoplasm, generative cell dividing, sperm cells and mature pollen formation, non observation of 3-nuclei structure.	Anthers shriveled of ‘Qx-115’ and ‘Kingfisher’.
Anther cracking and pollen dispersing stage	Anther cracking, pollen dispersing, very high degree of anther cracking, pollen dispersing completely.	‘Noa’: the degree of anther cracking inferior to ‘Qx-097’, pollen residue remains after pollen dispersing;
		‘Qx-115’: anther non-cracking, non-dispersal of pollen.

### Stamen anatomy during anther dehiscence of ‘Qx-097’, ‘Noa’, and ‘Qx-115’

**Figure 7:**
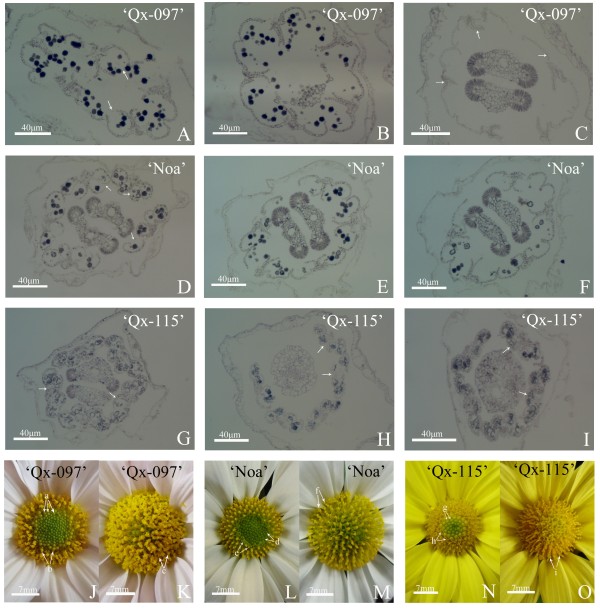
**Anatomical features of anther and Flower morphology during pollen dispersal of ‘Qx-097’, ‘Noa’ and ‘Qx-115’.** Pollen dispersing of ‘Qx-097’, ‘Noa’ and ‘Qx-115’. **(A, D, G)** The section of anthers just in pollen dispersal and anther cracking which corresponds to the anthers in disk florets a, d and g (arrows) in picture of material object. **(B, E, H)** The section of anthers at the stage of pollen dispersal a lot which corresponds to the anthers in disk florets b, e and h (arrows) in picture of material object. **(C, F, I)** The section of anthers after pollen dispersal which corresponds to the anthers in disk florets c, f and i (arrows) in picture of material object. **(J-O)** Flower morphology of ‘Qx-097’, ‘Noa’ and ‘Qx-115’ during pollen dispersing.

**Figure 8:**
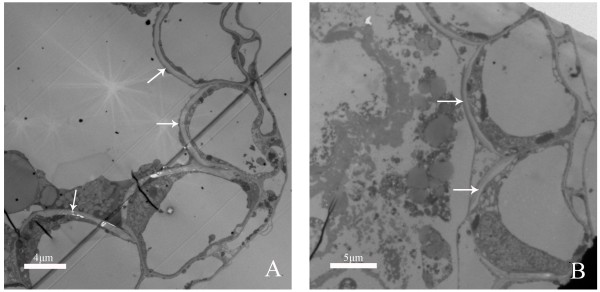
**Ultrastructure of endothecium layer cells at late stage of pollen development. (A)** ‘U’ shaped thickening of endothecium layer cells (arrows) in ‘Qx-097’. **(B)** ‘U’ shaped thickening of endothecium layer cells (arrows) in ‘Noa’.

The capacities of pollen dispersal are very different among ‘Qx-097’ , ‘Noa’ , and ‘Qx-115’ (Figure 7A-O; Table 2). ‘Qx-097’ has the highest capacity of pollen dispersal among three cultivars mainly, and no pollen grains were left in microsporangia of anther at the full stage of pollen dispersal (Figure 7A-C, J & K). ‘Noa’ has a relatively strong capacity of pollen dispersal, whereas its capacity of pollen dispersal is inferior to that of ‘Qx-097’ , for some pollen grains still remained in microsporangia of anther at the full stage of pollen dispersal (Figure 7D-F, L & M). ‘Qx-115’ does not have capacity of pollen dispersal, as it failed to disperse any pollen grains and pollen quantity in microsporangia of anther did not change much during the whole flowering process (Figure 7G-I, N & O). The capacities of pollen dispersal of the three cultivars are related to the degrees of anther cracking. ‘Qx-097’ has a highest degree of anther cracking, followed by ‘Noa’, whereas the anthers of ‘Qx-115’ are non-cracking and shriveled at late stage of flowering (Figure 7C, F & I). In addition, TEM images indicated that cell walls of endothecium layer in ‘Qx-097’ and ‘Noa’ thickened and became U-shaped at later stage of pollen development and the opening was toward the outer tangential wall (Figure 8A & B), which may the structure base of anther cracking and facilitate anther cracking. Therefore, the degree of anther cracking is one of the main factors affecting quantity of pollen dispersal of spray cut chrysanthemum through controlling the capacity of pollen dispersal. In other words, the level of anther cracking is positively related to quantity of pollen dispersal. For instance, ‘Qx-097’ is regarded as a cultivar with high pollen dispersal, one of the main reasons is that this cultivar has a very high degree of anther cracking during dehiscence (Figure 1G & H; Table 1). ‘Qx-115’ is a cultivar with more than fifteen thousand pollen per inflorescence, but does not disperse any pollen grains during anther dehiscence. The main reason is that anthers of ‘Qx-115’ fail to crack during dehiscence and remain intact (Figure 1K & L; Table 1).

## Discussion

Pollen is produced by plant stamen, the male reproductive organ of flower, and is very important for sexual reproduction of flowering plants [15,16]. However, pollen is often unwelcome in flower production, as the ornamental value of many flowers including chrysanthemum usually quickly decrease with the increase in quantity of pollen dispersal [17,18]. In addition, pollen can also create various allergic reactions in people [5,6,19,20]. In this study, four spray cut chrysanthemum cultivars are very different in the quantities of pollen dispersal. ‘Qx-115’ and ‘Kingfisher’ are cultivars with non-dispersing of pollen, ‘Qx-097’ is the cultivar with high quantities of pollen dispersal, and ‘Noa’ is the cultivar with less quantities of pollen dispersal (Figure 1, Table 1). The results presented here indicate that the significant differences in the quantities of pollen dispersal among the four chrysanthemum cultivars are largely due to two factors, pollen quantity per inflorescence and the capacity of pollen dispersal. In other words, the quantity of pollen dispersal is usually positively proportional to pollen quantity per inflorescence and the capacity of pollen dispersal.

Pollen quantity per inflorescence of spray cut chrysanthemum is jointly determined by pollen quantity per anther and the number of disk florets per inflorescence, but pollen quantity per anther is the main factor. Because the number of disk florets per inflorescence usually ranges from 100 to 200, whereas pollen quantity per anther has a wider range, usually 0-1000 (Table 1). There are many factors affecting pollen quantity per anther. For example, pollen quantity in the anther is influenced by the size of pollen sacs, the number of microspores mother cells, climate and nutrient conditions, and level of cultivation and management [15,21,22]. Liu *et al*. [23] and Tan *et al*. [24] thought that pollen quantity in anthers also depends on whether anthers develop normally. In the present study, we compared microsporogenesis and pollen development of the four cultivars with different quantities of pollen dispersal, and found that abnormalities often occurred during microsporogenesis and pollen development of ‘Kingfisher’, ‘Qx-115’, and ‘Noa’. For instance, nearly all the microspores mother cells degraded at the microsporocyte meiosis stage and asymmetrical dyad and tetrad in ‘Kingfisher’, which is the main reason why anther of this cultivar does not contain any pollen. However, abnormal phenomena were seldom observed in ‘Qx-097’ with lots of pollen grains in anther. Therefore, the reason for difference in pollen quantity per anther of the four chrysanthemum cultivars is mainly attributed to different levels of abnormalities occurring during microsporogenesis and pollen development. The possible reason for different levels of abnormalities is that long-term vegetative propagation of chrysanthemum, mainly cutting, has resulted in chromosome structure variation and abnormal meiosis. Similar phenomena were also observed in lily [25,26].

The capacity of pollen dispersal is another important factor influencing quantity of pollen dispersal in chrysanthemum, and is closely related to the speed and degree of anther dehiscence. The speed of anther dehiscence is the degree of anther dehiscence per unit time. We hypothesized that the higher the degree of anther dehiscence is, the stronger its capacity of pollen dispersal is. This hypothesis is confirmed by our results (Table 1). For example, ‘Qx-097’ has the highest degree of anther dehiscence among the four cultivars, and its pollen grains in anther disperse completely. ‘Qx-115’ does not disperse any pollen because its anthers do not crack at all, although its anther contains lots of pollen. The reason for anther abnormal cracking in ‘Qx-115’ may be the anther atrophy caused by pollen abortion and the anther walls of this cultivar has no special structure suitable for anther dehiscence. For ‘Qx-097’ and ‘Noa’, the cell walls in endothecium layer are ‘U’ shaped and thickened that may facilitate anther dehiscence, although the difference in degree of anther dehiscence between the two cultivars remains to be further investigated.

Anther dehiscence was once considered a simple process of tissue desiccation [27,28]. However, many studies showed that anther dehiscence is a complex process which is regulated by different mechanisms in different species [29-33]. For example, water channel protein, carbohydrate and K^+^ have been reported to be implicated in anther dehiscence by regulating cell osmatic potential which gave rise to dehydration of the tissue of anthers [34-36]. In addition, anther dehiscence was also regulated by hormones including jasmonic acid [37,38], auxin [30] and ethylene [39,40]. The main regulation model is that when anther tissue is under the regulation of hormones such as jasmonic acid etc., pollen grains and septum dehydrate at the right moment, and then K^+^ and secondary metabolites such as carbohydrate enter pollen grains and cause their rapid swell to produce the pressure on stomium under the help of water channel protein. Meanwhile, hormones such as ethylene promote the rupture of stomium cells by enzymatic hydrolysis to crack the anthers at last [29,31,33,41]. For instance, Sanders *et al.*[42] and Nagpal *et al.*[43] found that anthers of *Arabidopsis* mutant with defection in anther dehiscence could open after treatment with exogenous jasmonic acid and auxin. However, our results (data not shown) indicated that exogenous methyl jasmonate treatment couldn’t accelerate anther dehiscence of ‘Noa’ with incomplete dehiscent anthers and ‘Qx-115’ with non-dehiscent anthers, demonstrating jasmonic acid is possibly not a main factor affecting anther cracking in chrysanthemum. Moreover, external environment factors such as high temperature sometimes can also decrease the degree of anther dehiscence [44,45].

## Conclusion

In conclusion, we here performed a systematic study to investigate factors influencing quantity of pollen dispersal of four spray cut chrysanthemum cultivars with different quantity of pollen dispersal. Three findings are worth noting. Firstly, quantity of pollen dispersal in spray cut chrysanthemum are largely determined by pollen quantity per anther, and capacity of pollen dispersal. Secondly, significant differences in pollen quantity per anther among the four chrysanthemum cultivars are mainly attributable to significant differences in abnormalities occurring during microsporogenesis and pollen development. Thirdly, capacity of pollen dispersal is closely related to the degree of anther dehiscence. These important findings will provide valuable information for developing flower cultivars with less or no pollen dispersal in future breeding projects of chrysanthemum, even other crops, although the underlying mechanisms for pollen abortion and abnormal cracking of anthers remain to be further investigated.

## Competing interests

The authors declare that they have no competing interest.

## Authors’ contributions

NJT, XGW, FDC, JFJ and WMF designed the experiments. NJT, XGW, HBW, and YL performed the experiments. NJT, XGW, FDC and JFJ analyzed the data and wrote the manuscript. NJT and XGW revised the manuscript. All authors read and approved the final manuscript.
